# Pyrethroid Insecticide Resistance of Aedes albopictus and Aedes aegypti (Diptera: Culicidae) across the Hawaiian Islands

**DOI:** 10.21203/rs.3.rs-7303372/v1

**Published:** 2025-08-25

**Authors:** Sangwoo Seok, Miles T. McCollum, Christopher M. Jacobsen, Omar S. Akbari, Derrick K. Mathias, Yoosook Lee

**Affiliations:** University of Florida; University of Florida; Hawaii State Department of Health; University of California; University of Florida; University of Florida

**Keywords:** Aedes, insecticide resistance, resistance mutation, Hawaii

## Abstract

**Background:**

*Aedes albopictus* (Skuse, 1894) and *Aedes aegypti* (Linnaeus, 1762) (Diptera: Culicidae) are invasive species in the Hawaiian Islands as well as other islands of the Pacific and serve as the primary vectors of arboviruses like dengue virus. Despite its significance to public health, data on their insecticide resistance remains limited. Knowledge of the level of insecticide resistance is critical in developing effective mosquito control strategies, especially when an arboviral disease outbreak occurs.

**Methods:**

This study investigated the phenotypic and genotypic resistance of Hawaiian *Ae. albopictus* and *Ae. aegypti* to permethrin, one of the commonly used pyrethroids. Genomic sequences of 93 *Ae. albopictus* samples from four Hawaiian Islands (Kaua’i, O’ahu, Maui, and the Island of Hawai’i) were screened for non-synonymous mutations in the voltage-gated sodium channel (VGSC) gene (chromosome 3: 345,142,499 − 345,663,668). Phenotypic resistance to permethrin was assessed using a modified Centers for Disease Control and Prevention (CDC) bottle bioassay on *Ae. albopictus* and *Ae. aegypti* collected from two socio-environmentally distinct sites.

**Results:**

Among 4,101 single-nucleotide polymorphisms (SNPs) identified in the VGSC region of *Ae. albopictus* genomes from for Hawaiian Islands, 61 were classified as synonymous. No non-synonymous mutations were found, suggesting an absence of genotypic resistance to pyrethroids in these populations. In phenotypic assays, over 97% of *Ae. albopictus* and all *Ae. aegypti* individuals were knocked down within 10 minutes of permethrin exposure. These high knockdown rates indicate that both species remain phenotypically susceptible to permethrin.

**Conclusions:**

This study is the first study reporting the phenotypic insecticide resistance profile of Hawaiian *Aedes* mosquitoes. Hawaiian populations of *Ae. albopictus* and *Ae. aegypti* remain susceptible to pyrethroids, as demonstrated by the absence of VGSC mutations and high knockdown rates in permethrin bioassays. While no genotypic and phenotypic resistance was detected in these two *Aedes* species, monitoring for resistance in other mosquito species and through alternative mechanisms is needed.

## Background

Among 119 *Aedes* species found in the Pacific Islands, *Aedes albopictus* and *Aedes aegypti* are recognized as the main vectors of arboviruses, including dengue, chikungunya, and Zika viruses [1]. They have spread across the Pacific Islands, and, notably, the Hawaiian Islands were one of the first documented regions in the world to report the invasion of these two *Aedes* mosquito species [2, 3]. These two *Aedes* mosquitoes invaded Hawaii before 1900, and since then, dengue outbreaks caused by both species have been reported [3].

The widespread distribution of these *Aedes* mosquitoes and their critical involvement in arbovirus transmission emphasize the necessity of evaluating their susceptibility to insecticides. Despite being major vectors in the Hawaiian Islands for a long time, there are no available publications on the insecticide resistance of *Aedes* mosquitoes from the region, except for limited references to a few individuals in global-scale research investigations [4]. The presence and development of insecticide resistance in mosquito populations can undermine the effectiveness of chemical products used for mosquito control [5].

Pyrethroids have constituted the majority in terms of quantity of usage among the four classes of insecticides approved by the World Health Organization for the control of disease vectors between 2000 and 2019 [6, 7]. Pyrethroid insecticides target voltage-gated sodium channels (VGSC), which are formed by four domains, each comprising six segments [8]. The binding of pyrethroids to VGSC results in the channels remaining open for an extended period of time, disrupting nerve function. Consequently, mutations in the VGSC gene interfere with the binding of pyrethroids, thereby conferring knockdown resistance (*kdr*) to pyrethroids to *Aedes* [9], *Anopheles* [10], and *Culex* [11] mosquitoes.

Non-synonymous mutations in the VGSC gene alter the amino acid sequence of the channel protein, thereby modifying its structure. The level of resistance conferred by such mutations can vary depending on their location on the VGSC gene [12]. Mutations occurring near the pyrethroid-binding site can hinder the attachment of the insecticide and confer resistance. The level of resistance may be further enhanced due to interactions between mutations when certain mutations occur in combination [9, 13]. The presence of such compound mutations has been frequently observed in field studies, which can be interpreted as a consequence of evolutionary selective pressure in environments where multiple insecticides are used. As resistance to pyrethroids increases, the effectiveness of existing insecticides decreases [9], highlighting the need for alternative chemicals or novel control strategies.

The phenotypic resistance of Hawaiian *Aedes* mosquitoes to pyrethroids has never been reported [4, 14]. Available literature about genotypic resistance for the Hawaiian *Ae. albopictus* population is limited to a single study, which reported the absence of the resistant F1534 mutation in O’ahu, Hawaii [15]. Similarly, in the Hawaiian *Ae. aegypti* collected from the Islands of Hawai’i, no resistance that confers mutations has been identified [16]. On the other hand, the consistent detection of mutations in the VGSC gene of other insects, such as bed bugs, suggested the persistent use of pyrethroids in O’ahu, Hawaii [17], which could contribute to the development of pyrethroid resistance in Hawaiian mosquitoes if mosquitoes come indoors to bite people and come in contact with insecticide-treated surface. Given the limited data, further genetic surveillance and phenotypic characterization are needed to understand the insecticide resistance profiles of these populations. Such knowledge is crucial for devising mitigation strategies in genetic control trials and managing unforeseen circumstances that could compromise control effects [18].

In this study, we explored single-nucleotide polymorphisms (SNPs) in the coding DNA sequence (CDS) of VGSC to determine whether Hawaiian *Ae. albopictus* populations possess resistant mutations or not. We further conducted Centers for Disease Control and Prevention (CDC) bottle bioassays on Hawaiian *Ae. albopictus* and *Ae. aegypti* populations to characterize their phenotypic resistance levels to permethrin, a commonly used pyrethroid insecticide. Understanding the insecticide resistance profile will help in establishing effective vector management and control strategies.

## Methods

We used 93 genomic sequences of the Hawaiian *Ae. albopictus* population (SRA Accession number: SAMN472862276-SAMN47286368) (Table S1), which were obtained from our previous study [19] to identify potential non-synonymous mutations in the VGSC region. These samples were collected from four Hawaiian Islands: Kaua’i, O’ahu, Maui, and the Island of Hawai’i. Variant calling was performed using Freebayes version 1.3.6 [20] based on the *Ae. albopictus* reference genome AalbF5 (GCF_035046485.1). The analysis focused on the VGSC region located on chromosome 3, spanning positions 345,142,499 − 345,663,668. Missing data was allowed up to 10%. To identify mutations within the CDS of the VGSC region, we examined SNPs in this region. Processing and filtering of SNPs were carried out using BCFtools version 1.13 [21] and VCFtools version 0.1.16 [22]. Indels were excluded, and only variants with a read depth between eight and 120 and a quality score of 30 or higher were retained. Visualization and confirmation of SNPs were conducted using the Integrative Genomics Viewer (IGV) version 2.18.4 [23], where identified variants were compared against the reference genome.

**Figure 1:**
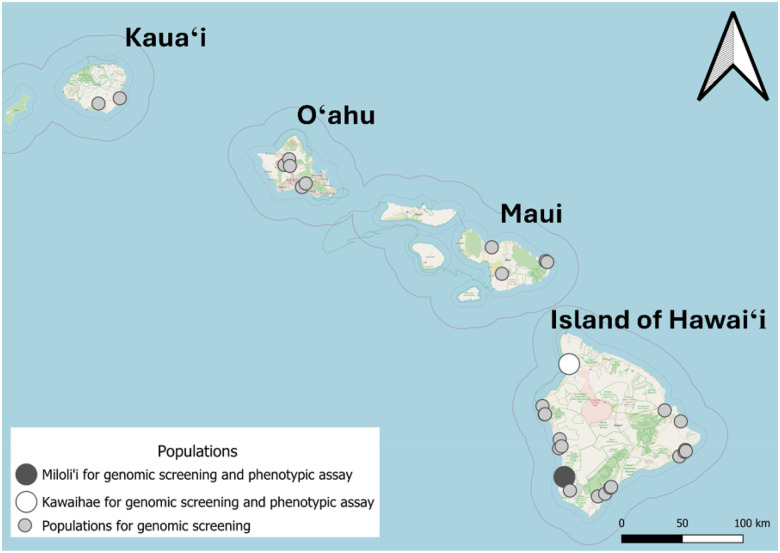
Locations of *Aedes* mosquito populations analyzed via genome screening and phenotypic assay across four Hawaiian Islands. Populations represented by dark gray (Miloli i) and white (Kawaihae) circles were included in both genomic screening and phenotypic assay. Light gray circles indicate populations included in genomic screening only. This map was created using the Free and Open Source QGIS.

For phenotypic insecticide resistance assay, eggs of *Ae. albopictus* and *Ae. aegypti* were collected from Kawaihae and Miloli’i on the Islands of Hawai’i (Fig. 1). Kawaihae is a coastal town known for its harbor, functioning as a regional commercial port. Miloli’i is a coastal fishing village located over 3 km from the main road, where residents use rainwater catchment systems for their water supply. These two sites were selected to investigate variation in insecticide resistance between populations exposed to different socio-environmental settings. Collecting was conducted from February to March of 2023, using oviposition traps, filled with a seven-day-old grass infusion prepared by steeping 160 g of Guinea grass (*Megathyrsus maximus*) in 19 L of water [24]. Subsequently, eggs were transferred to the laboratory of the Hawaii Department of Health in Hilo, Hawaii, for rearing and storage, following procedures outlined by the PacMOSSI (Pacific Mosquito Surveillance Strengthening for Impact) consortium [25]. After conditioning and drying, eggs were kept in sealed plastic containers (30 cm [L] × 15 cm [W] × 11 cm [H]; Sterilite) until used for hatching. In April and May, the eggs were placed in a 25 cm [L] × 20 cm [W] × 4 cm [H] tray with tap water, which had been boiled and cooled to ambient temperature. The larvae fed ground fish food (TetraMin; Tetra, VA) hatched and developed into pupae in approximately seven days. Pupae were transferred into 12 cm-diameter, plastic cups (946 ml; Taral Plastics, CA) and then placed in mosquito-rearing cages (30.5 cm [L] × 30.5 cm [W] × 30.5 cm [H]; BioQuip 1450B; company is out of business and its location information no longer available.). Cotton balls wet with 10% sugar solution were provided on top of the rearing cages for the emerging adults. The temperature in the rearing environment was 25.0 ± 0.6 °C (M ± SD), and the humidity was 68.8 ± 4.0%. Both female and male adults that were 3–8 days old were used for the bottle bioassay.

A CDC bottle bioassay was conducted to test phenotypic resistance to permethrin in two Hawaiian populations of the two species. We followed the CDC protocol [26] with a minor modification in which only one individual was introduced into each test bottle to assess the resistance of individual mosquitoes, and 25–30 individuals were introduced into a control bottle. This allows correlation to be made between the phenotype data and genotype data for each individual in subsequent analysis. This approach has been used in *Ae. aegypti* research [9, 27] to uncover relationships between phenotype and genotypes. A stock solution of permethrin provided by the CDC was diluted in acetone to a concentration of the diagnostic dose (43 μl/ml). Thirty test bottles were coated with 1 ml of the diluted permethrin solution, while a control bottle was coated with 1 ml of acetone. Their knockdown was recorded at 5, 10, 15, 20, 30, 45, 60, 90, and 120 minutes. The diagnostic time was 10 minutes of exposure [26].

Statistical analyses were conducted to evaluate differences in survival rates across species and populations. Log-rank tests were applied to survival data recorded at 5 and 10 minutes to identify significant differences between species and populations. These time points were selected as 10 minutes of continuous exposure to permethrin, which is the diagnostic time for these two species [26]. Analyses were conducted using Python 3.10 with the lifeline library version 0.27.8 [28]. Bonferroni corrections were applied for multiple comparisons.

## Results

In the VGSC region of the *Ae. albopictus* genome, a total of 4,101 SNPs were identified across the 93 genomic sequences from four Hawaiian Islands. Within the CDS region, 61 synonymous mutations were observed. Notably, no non-synonymous mutations were found, indicating an absence of amino acid changes in the VGSC region of the Hawaiian *Ae. albopictus* populations.

**Figure 2:**
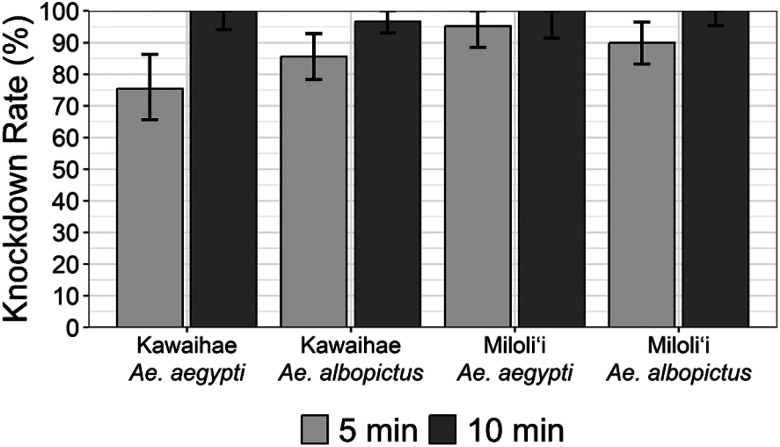
Knockdown rates of *Ae. aegypti* and *Ae. albopictus* mosquitoes collected from Kawaihae and Miloli’i in the Island of Hawai’i at 5- and 10-min exposure to permethrin (43 μl/bottle).

A total of 169 *Ae. albopictus* and 102 *Ae. aegypti* were tested using the modified CDC bottle bioassay, with 90 *Ae. albopictus* and 61 *Ae. aegypti* collected from Kawaihae, and 79 *Ae. albopictus* and 41 *Ae. aegypti* from Miloli’i. All *Ae. aegypti* from Kawaihae and Miloli’i were knocked down at 10 minutes, indicating the susceptible population according to the criteria provided in [26]. The mortality rate of *Ae. albopictus* from Kawaihae and Miloli’i were 96.7% and 100% at 10 minutes, respectively (Fig. 2). It indicates that the two *Ae. albopictus* populations are susceptible to permethrin. The mortality rate of the Kawaihae *Ae. albopictus* population reached 100% at 15 minutes of exposure. On the other hand, none of the control *Aedes* mosquitoes were dead at the diagnostic time.

Statistical analyses revealed that the knockdown time of Kawaihae *Ae. aegypti* population significantly differed from that of the Miloli’i *Ae. aegypti* (adjusted *P* < 0.001). However, no significant differences were found between Kawaihae *Ae. albopictus* and Miloli’i *Ae. albopictus* (adjusted *P* = 0.24), between Kawaihae *Ae. albopictus* and Kawaihae *Ae. aegypti* (adjusted *P* = 0.56), and between Miloli’i *Ae. albopictus* and Miloli’i *Ae. aegypti* (adjusted *P* = 0.25).

## Discussion

The screening of non-synonymous mutations in the VGSC gene of Hawaiian *Ae. albopictus* populations indicated that this species has not developed target site insensitivity mutations yet. The samples utilized in genetic screening were obtained from a broader range, including Kaua’i, O’ahu, Maui, and the Island of Hawai’i, than the samples used for the modified CDC bottle bioassay. This suggests that the susceptibility to pyrethroids is not confined to the Island of Hawai’i populations but could extend across *Ae. albopictus* populations throughout the Hawaiian Islands. The lack of standing genetic variations in the VGSC among Hawaiian populations could minimize the potential for insecticide resistance to rise via selection on standing variations, although other forms of mutations, such as de-novo mutations [29], are possible. Adaptive introgression [30, 31] is also possible but unlikely in Hawaii because no other *Aedes* species occur there that could hybridize and produce viable offspring.

This study is the first study reporting the phenotypic insecticide resistance profile of Hawaiian *Aedes* mosquitoes. All phenotypic insecticide resistance tests by populations and species showed that Hawaiian *Ae. albopictus* and *Ae. aegypti* populations are susceptible to permethrin. Statistical analyses showed Kawaihae *Ae. aegypti* are significantly different from the Miloli’i population. The significant difference appears to be due to the relatively lower mortality rate of Kawaihae *Ae. aegypti* population when exposed to permethrin for five minutes. However, it does not indicate that the population is resistant to permethrin, as their mortality rate eventually reached 100% at 10 minutes of exposure.

Since previous studies on insecticide resistance mutations in Hawaiian *Aedes* mosquitoes have only focused on a limited number of genes [15, 16], we cannot rule out the presence of other resistance-associated mutations. Pyrethroid resistance relies not only on VGSC mutations but also on metabolic resistance mechanisms, including detoxification by cytochrome P450 monooxygenases [32, 33]. Therefore, further studies should investigate pyrethroid resistance that may have developed through other mechanisms, as well as resistance to other classes of insecticides, such as organophosphates or carbamates, to provide a comprehensive potential resistance profile for Hawaiian mosquitoes.

We adopted a modified CDC bottle bioassay to perform insecticide resistance testing at an individual level. This method allows us to analyze the relationship between phenotypic insecticide resistance and genotypic insecticide resistance [9, 27]. However, since no individuals with phenotypic resistance were identified in this study, we did not investigate potential mutations associated with genotypic insecticide resistance from the sampled we used for the CDC bottle bioassay.

While this study highlights the susceptibility of Hawaiian *Ae. albopictus* and *Ae. aegypti* populations to permethrin, additional research is needed to monitor insecticide resistance in other Hawaiian mosquito species, such as *Ae. japonicus* and *Wyeomyia mitchellii*. There are indications that Hawaiian populations may have a chance to develop insecticide resistance. Mutations in the VGSC gene have been observed in several *Ae. aegypti* populations in the PICTs, such as Vanuatu, Kiribati, New Caledonia, and Fiji [4] Given the high invasiveness of *Ae. albopictus* and *Ae. aegypti* [1, 4], the insecticide-resistant population could be introduced and established if early detection and adequate response are not implemented in these islands. In addition, in the O’ahu region, VGSC mutations in bed bugs were consistently detected in 2009 and 2019, alongside the emergence of new mutations in 2019 [17]. It indicated that the persistent use of pyrethroid insecticide in Hawaii could exert selection pressure for insects to develop resistance under increased insecticide exposure. Even if this insecticide is not specifically targeted at mosquitoes, it may still contribute to the development of insecticide resistance in mosquito populations [34]. On the other hand, given the high volume of tourism in Hawaii, it is also possible that bed bugs with insecticide resistance were introduced from outside Hawaii rather than developing their resistance locally. Moreover, mosquito biting could take place predominantly outdoors in Hawaii, reducing the frequence of direct contact with insecticide-treated surface indoors for bedbug treatment. It may explain why insecticide resistance has not been observed in *Aedes* mosquitoes in this study.

## Conclusion

This study is the first study reporting the phenotypic insecticide resistance profile of Hawaiian *Aedes* mosquitoes. In this study, we found no evidence of pyrethroid resistance in Hawaiian populations of *Ae. albopictus* and *Ae. aegypti*, demonstrating their susceptibility to permethrin based on both genotypic and phenotypic analyses. Therefore, the use of permethrin remains viable option for *Aedes* control in Hawaii. The absence of any mutations in the VGSC gene and the lack of phenotypic resistance observed through modified CDC bottle bioassay suggest that these mosquito species and populations have not yet developed resistance to pyrethroids. Given that the genetic screening included samples from multiple Hawaiian Islands, this susceptibility may be widespread across the Hawaiian Islands. These findings underscore the effectiveness of permethrin in controlling Hawaiian *Aedes* mosquitoes. Expanding resistance monitoring to include other mosquito species and mechanisms will be crucial to sustaining effective vector control strategies in Hawaii.

## Supplementary Material

This is a list of supplementary files associated with this preprint. Click to download.

- TableS1PV.xlsx

## Data Availability

Data is provided within the manuscript as a supplementary information file.
